# Cases Illustrative of Criminal Abortion

**Published:** 1859-05

**Authors:** Horatio R. Storer

**Affiliations:** Boston


*Cases Illustrative of Criminal Abortion.* By HORATIO R. STORER, M. D., of Boston. (Read before the Boston Society for Medical Observation, Feb. 7, 1859.)

CASE I. Mrs. H—, of Roxbury, applied to me for treatment, on the 14th of October last. Patient, an American by birth, though the wife of a German, is a well-formed, healthy looking woman, some twenty years of age, and was then five and a half months gone with first child. Her general health had been good, and till the present she had never suffered from any form of neuralgic pain.

She reported excessive toothache, of nearly two months' standing; that it commenced on the left of the lower maxilla, but then affected both sides of both jaws; that during the whole period she had been under the charge of a physician, and had been thoroughly and actively treated, by anodynes, local and general, by antispasmodics, purgatives, fomentations, counterirritants; that a tooth, apparently the only carious one she had, had been extracted ten days previously, and that it had been proposed to remove others, to which, however, she would not consent—all without the slightest relief.

She alleged, and showed, loss of sleep and of appetite, great general prostration, excessive despondency of mind. After the extraction of the tooth, abortion had threatened—and she now begged that it might be brought on; declaring, if refused, that she would induce it upon herself, rather than endure further pain.

She was ordered a fragment of pellitory root, pyrethrum, as a direct gingival stimulant, though horseradish would probably have answered the purpose, and on the second day presented herself cured. There has been no return of the malady, save a slight attack on January 19th which was readily relieved by the same tretment. Patient was confined on February 3d; and is doing well.

I report this case for two reasons. In the first place, as an instance of the frequent success of simple and apparently trivial remedies after severe ones have failed. The affection seems to have been entirely neuralgic in its character, reflex, the result of the uterine irritation.—All other causes mentioned by writers as liable to produce it were absent; there was no local inflammation, no general catarrhal affection; the disorder did not commence at, and apparently was in no way dependant upon, the carious tooth, at least it was not relieved by its removal, nor by the local blood-letting then occasioned.

I am aware that sialagogues, according to Gardien, and he is apparently endorsed by Churchill, are supposed indicated only in those instances where the toothache is in consequence of a general catarrhal affection, which did not here exist; but on the other hand their use would seem, on the simplest theoretical grounds, among the first procedures that would occur to the mind.

The second of the reasons referred to is the following: that I may express my strong disapproval of the practice, still extensively obtaining among physicians and dentists, of subjecting patients to the risk of miscarriage, which must be confessed excessive, by the extraction of teeth during pregnancy. This procedure should in no instance be resorted to till every other measure which affords any prospect of relief has been faithfully employed. In the history reported, it is seen that such was not the case.

Extraction has been recommended by authorities who are respected, by Campbell, Gardien, Capuron and others, on the supposition that there is a greater liklihood of abortion from the continued pain; but against this argument I place the facts that after resisting many remedies, the pain often disappears spontaneously—as is indeed allowed by one of the writers instanced, Capuron—and that in more plausible measures, tried and untried, readily occurring upon reflection to all who do not blindly follow the books, there is, I think, a greater probability of success. Anæsthesia, local and general, have both been found to avail. I would suggest, as worthy attention, a modification of the process of subcutaneous injection, proposed by Alexander Wood, of Edinburgh, and so successfully employed; merely here introducing the opiate beneath the mucous membrane, which I am not aware to have yet been done.* Should this also fail, a direct topical application, either of a local anæsthetic or a gentle stimulant, might be made to the cervix uteri; but as this latter procedure, though at times successful in the obstinate vomiting of pregnancy, cannot be used with too much caution during gestation, would, therefore, be seldom justifiable; and only in the cases where the extraction of teeth in pregnant woman can ever be defended, those, namely, where abortion is actually threatening and apparently at hand.

It might be asked how the case now reported can fairly and with

* After this paper was in type, I find, by the Edinburgh Medical Journal for Nov. 1858, p 424, that the above suggestion has been part.ally anticipated; a dentist, Dr. Smith, having had recourse to the measure, for the painless extraction of teeth. The experiment does not yet seem to have been tried, however, as here proposed, with a view to prevent that operation.

justice be considered as in any way illustrative of the subject of criminal abortion, because, though menaced and solicited, this did not actually take place. It is an acknowledged principle of jurisprudence that a person must be presumed to intend all the natural, probable, and usual consequences of his own act.* Whenever, by any operative or other procedure, a physician directly produces abortion, unintentional though this may be, if in the absence of any precaution that might have been taken, he must be considered, to the extent evidenced by the history of the patient, responsible therefor; and the class of cases to which that now reported belongs, is accordingly open to as legitimate a question of obstetric morality and of criminal responsiblity as that other series, of late so ably discussed by Dr. Churchill, of Dublin.†

Case II. I was called to attend Mrs. S—, of Plympton Court, on the afternoon january 2d, 1859 and found her flowing profusely, this having continued for many hours; the attacks at first being considered menorrhagic, as she was still suckling, till an abundant escape of coagula rendered probable its true character.

Upon examination, the uterus proved somewhat enlarged and slightly retroverted, the os was with difficulty reached, sufficiently patulous to admit the tip of a single finger, but its outline laterally and symmetrically fissured, so that the cervix presented to the touch an apparently deep and incised transverse wound completely through its substance, with irregular edges and indentations simulating artificial punctures. These irregularities and depression were not like those presented by malignant diseases; there was no fetor or other usual or plausible symptom of such, save the hemorrhage. Within the os a mass was felt presenting, which, from the absence of other signs of polypus, the sudden and profuse sanguineous discharge, and the faint uterine contractions that from time to time occurred, conjoined as these were with a contained body of hardly sufficient size, if a polypus, to have itself excited them, I did not hesitate to pronounce a partially detached ovum.

My first impression from the physical examination was of course that the abortion must have been owing to direct instrumental or other violence, which, however, the patient persistingly denied. Upon learning that she was of the Catholic faith these suspicions were somewhat

* Davis, Crim. Justice, 483.

† Dublin Quarterly Journal of Med. Science, August and November, 1858.

allayed; for reasons I shall elsewhere fully set forth, and which are borne out by statistics so far as existing, and by general experience.

I was the further convinced of my error upon hearing the past history of the case. The patient, as already remarked, was nursing—sixteen months having elapsed since her last confinement. The catamenia had not returned, and the only possible cause she had had for supposing she might be pregnant, beyond the ordinary exposure of married women, was an apparent slight decrease in the amount of milk secreted; of the existence of this decrease, however, she was by no means certain, and had attributed to it no importance. She had strained herself within a day or two, by reaching to a shelf.

Mrs. S— had been confined four times; in every instance labour being exceedingly tedious, and delivery accomplished by instrumental aid. Though the children were all born living—their extraction was effected with much difficulty, and the application of considerable force. I could not doubt, therefore, that laceration of the cervix had occurred at one or more of the times referred to, sufficient to account for all the appearances that were now observed; an accident by no means incompatible with the exercise on those occasions of all needful caution and skill, for it is notorious that lesions, to a greater or less extent, of the margin of the os are extremely common, even in natural labors.

In the treatment of the present case there was nothing unusual. It seemed impossible to check the abortive process, and there being little danger from confined internal hemorrhage in early miscarriage, when the uterine cavity is but slightly enlarged or dilatable, the vagina was plugged; strips of cotton being used, inserted separately and tightly packed, like the foil in the process of tooth-filling. Upon their removal, three hours after, the os was found widely patulous, proper forceps were introduced, and an embryo, some six weeks advanced, was removed; upon which the hemorrhage at once entirely ceased.—The patient has made a good recovery.

January 28th, four weeks subsequent to the miscarriage, the physical signs presented by the uterus were still persistent, as described above.

Upon reviewing this case, I think it important to dwell upon the diagnostic peculiarities it presents, unadverted to, so far as I am aware, by any obstetric writer. We are all familiar with the various permanently disastrous results at times following instrumental or otherwise difficult labour, the adhesions, bands, and fistulæ that not unfrequently come under medical observation; but to find such, in the absence of all signs of concomitant or consequent malignant disease, and accompanied by profuse hemorrhage—their edges, the cicatrices themselves, and the depressions between them obscured and filled by coagula, and at the same time, and in the midst of these physical anomalies, the presentation of an aborting ovum—would raise, I may surely say, in almost every mind the suspicion of foul and criminal interference. Were death to occur under such circumstances, the result at an inquest could hardly be doubted, unless unusual care were observed at the autopsy to remove by ablution all clots obscuring the age of the existing lesions; a precaution that in most instances would hardly be observed, for fear of disturbing any attachments of the ovum—so often in these cases preserved for the cabinet—that might still obtain.

It is my duty to lay the more stress upon this case and its several suggestions lest at other times, and in another connection,* any remarks of my own might seem unjust. But, on the other hand, I am thus strengthened in my belief that many of the medico-legal relations of criminal abortion are as yet uninvestiagted or unfound, and that with every step towards their elucidation an advance is made towards the ultimate suppression of the crime.—*American Journal of the Medical Sciences.*

---

* Essay on Criminal Abortion. North Amer. Med.Chir. Review, May, 1858.

